# Preregistered Direct Replication of “Sick Body, Vigilant Mind: The Biological Immune System Activates the Behavioral Immune System”

**DOI:** 10.1177/0956797620955209

**Published:** 2020-10-20

**Authors:** Joshua M. Tybur, Benedict C. Jones, Lisa M. DeBruine, Joshua M. Ackerman, Vanessa Fasolt

**Affiliations:** 1 Department of Experimental and Applied Psychology, Vrije Universiteit Amsterdam; 2 Institute of Brain and Behavior Amsterdam, The Netherlands; 3 Institute of Neuroscience & Psychology, University of Glasgow; 4 School of Psychological Sciences and Health, University of Strathclyde; 5 Department of Psychology, University of Michigan

**Keywords:** evolutionary psychology, threat, attention, disgust, health, open data, preregistered

## Abstract

The tendency to attend to and avoid cues to pathogens varies across individuals and contexts. Researchers have proposed that this variation is partially driven by immunological vulnerability to infection, though support for this hypothesis is equivocal. One key piece of evidence (Miller & Maner, 2011) shows that participants who have recently been ill—and hence may have a reduced ability to combat subsequent infection—allocate more attention to faces with infectious-disease cues than do participants who have not recently been ill. The current article describes a direct replication of this study using a sample of 402 individuals from the University of Michigan, the University of Glasgow, and Vrije Universiteit Amsterdam—more than 4 times the sample size of the original study. No effect of illness recency on attentional bias for disfigured faces emerged. Though it did not support the original finding, this replication provides suggestions for future research on the psychological underpinnings of pathogen avoidance.

Across taxa, animals possess a number of behavioral adaptations that function to mitigate the costs imposed by infectious microorganisms (Hart, 2011). Humans are no exception. For example, our visual and olfactory systems have evolved a sensitivity to cues related to infectious agents, such as the textures and colors associated with other people’s immune responses (e.g., pus) and the chemical by-products of bacterial presence on corpses (e.g., putrescine). When these cues to pathogens are detected, facial movements and motivational tendencies are deployed in a manner tailored to avoiding pathogens (Tybur, Lieberman, Kurzban, & DeScioli, 2013). Our pathogen-avoidance adaptations have consequences for myriad phenomena traditionally under the purview of social, developmental, cognitive, and clinical psychologists, including food learning, stigmatization, hygiene ruminations, and mate choice (for reviews, see Ackerman, Hill, & Murray, 2018; Curtis, 2014; Murray & Schaller, 2016; Schaller & Park, 2011; and Tybur & Lieberman, 2016). Consequently, a large body of research—what has come to be known as the *behavioral-immune-system literature*—has focused on better understanding these adaptations.

A sizeable proportion of work on the behavioral immune system has been designed to better understand why people vary in their pathogen-avoidant behaviors. Much of this research has been inspired by the evolutionary-biology literature, which highlights the fact that organisms neutralize pathogens not only through avoidance but also through immunological resistance (e.g., via proinflammatory cytokines) and tolerance (Medzhitov, Schneider, & Soares, 2012). Given that investment in these strategies might be traded off against each other (Gangestad & Grebe, 2014), researchers have proposed that a decreased ability to resist infection leads to greater investment in avoiding infection (e.g., Oaten, Stevenson, & Case, 2009). Multiple studies have presented data that have been interpreted as supporting this prediction: Putatively lower immunological resistance was associated with (a) greater skin conductance response while participants viewed visual cues to pathogens (Ersche et al., 2014), (b) higher reported disgust toward such images (Fleischman & Fessler, 2011), and (c) more reported anxiety toward infection-risky behaviors (Oaten, Stevenson, & Case, 2017). These studies were limited by their measurement of capacity to resist infection, however; they treated cocaine dependency, progesterone, and affliction with rheumatoid arthritis, respectively, as proxies for decreased immunological resistance.

To the best of our knowledge, the most compelling evidence for a relation between immunological resistance and pathogen avoidance in humans was provided by Miller and Maner (2011), who, in Study 1, found that participants who had recently been ill—compared with participants who had not—displayed heightened attention toward cues to pathogens. Specifically, participants completed a dot-probe task, in which they were asked to identify a target object as either a circle or a square as quickly as possible. In critical trials, faces that were either typical (referred to as “healthy” in the original study) or disfigured appeared shortly before the target object in a different screen quadrant. Higher reaction times during these critical trials were interpreted as reflecting greater attention toward the distractors. Analyses of reaction times revealed that illness recency interacted with distractor type: The 28 participants who had recently been ill took 38 ms longer to identify the targets after disfigured-face distractors relative to typical-face distractors (*SD* = 65.25, *d*_z_ = 0.58), whereas reaction times did not differ across distractor face types for the 66 participants who had not recently been ill (mean difference = 4 ms faster to identify targets after disfigured-face distractors, *SD* = 58.32, *d*_z_ = 0.07; J. K. Maner, personal communication, April 25, 2018).

The use of illness recency to test behavioral-immune-system hypotheses offers a clear advantage over the other approaches described above. Illness recency is presumably less confounded with other variables than are cocaine dependency or rheumatoid arthritis, and it has clearer effects on immunological resistance than does progesterone. Indeed, immunologists have noted that people are more likely to die of secondary bacterial infections after influenza than from the viral infection itself, and research exploring this phenomenon has found that resisting influenza raises anti-inflammatory interleukin-10 and depresses natural killer cells (Small et al., 2010; Van der Sluijs, 2004). Both of these effects compromise the ability to resist subsequent infections.

The finding that illness recency affects attention to pathogen cues has influenced theoretical models of the behavioral immune system (e.g., Ackerman et al., 2018; Murray & Schaller, 2016; Tybur & Lieberman, 2016), and it has been showcased as a key example of the relation between immunological resistance and the behavioral immune system (e.g., Fleischman & Fessler, 2018; cf. Jones et al., 2018b). The current study directly replicated Study 1 of Miller and Maner (2011) to test the robustness of the effect of illness recency on attention to pathogen cues. It had an additional goal. Disgust-sensitivity instruments, in which participants report their disgust toward disgust-eliciting situations, have been criticized as potentially insensitive to short-term fluctuations in pathogen-avoidance motivations (Fleischman & Fessler, 2018; Tybur, Çınar, Karinen, & Perone, 2018). Self-reported disgust toward visual cues to pathogens has been proposed as a more sensitive measure of such fluctuations (Fleischman & Fessler, 2018). Therefore, we also assessed whether scores on a disgust-sensitivity instrument and self-reported disgust toward visual cues to pathogens are related to illness recency. Note that we administered these additional measures, which were not included by Miller and Maner (2011), after the materials collected in the original study. Consequently, their presence did not interfere with the replication.

This replication attempt comes at a critical time for the behavioral-immune-system literature. Although the studies summarized above have pointed to a relation between immunological resistance and avoidance, others have found no relation between progesterone and disgust sensitivity (Jones et al., 2018a), infection history and disgust sensitivity (de Barra, Islam, & Curtis, 2014), and ecological pathogen stress and disgust sensitivity (Tybur et al., 2016). These studies used large samples (all had more than 280 participants, which would afford more than 90% power to detect an effect size [*r*] of .20 for a between-subjects design), and Jones et al. also collected repeated assessments of progesterone and disgust sensitivity within participants. Hence, these null results cannot easily be dismissed as Type II errors. Ultimately, given the combination of positive and null results, some doubts regarding the relation between immunological resistance and pathogen avoidance persist. Hence, replicating one of the key results supporting this relation could pay dividends in future theory development in this area. Further, measuring both attention to pathogen cues and disgust sensitivity indicates whether inconsistent results within this literature reflect differences in phenomena measured (e.g., visual attention vs. disgust sensitivity).

Ultimately, then, the present work (a) directly replicates a key study in the behavioral-immune-system literature and, in doing so, (b) informs the degree to which illness recency relates to visual attention to pathogen cues versus self-reported disgust (via semantic and visual descriptions) toward stimuli connoting heightened infection risk.

## Method

Except where noted, all methodological details—including all stimuli and dot-probe procedures—and all analyses were identical to those used by Miller and Maner (2011, Study 1). All procedures from the original study (e.g., Inquisit files, stimuli, trial order, and order of questionnaires) were confirmed with the senior author from the original study (J. K. Maner, personal communication, April 25, 2018).

### Participants

Miller and Maner (2011) tested 96 participants (all between 18 and 30 years of age). Simonsohn’s (2015) “small-telescopes” approach to sample size suggests that replications should have at least 2.5 times the sample size of the original study (hence, a replication sample size of 240). Given the potential of randomly recruiting a lower proportion of recently ill participants (relative to the sample in the original study), we preregistered a sample size of 360 participants. We ultimately enrolled 413 participants between the ages of 18 and 30 years across the three test sites (*n* = 147 at Vrije Universiteit Amsterdam, *n* = 145 at the University of Michigan, and *n* = 121 at the University of Glasgow). According to an analysis in G*Power (Version 3.9.1.7; Faul, Erdfelder, Buchner, & Lang, 2009), this sample size affords more than 99% power to detect an interaction effect (*d*) of 0.65 (equivalent to that reported in the original study). This calculation was based on an exclusion rate of 2% (see below), categorization of 30% of the sample as recently ill (i.e., 121 participants who had recently been ill and 283 participants who had not), and setting α to .025 (see below). Given evidence that experimenters’ expectations can influence results (Gilder & Heerey, 2018), experimenters were blind to study hypotheses.

### Stimuli

We used 40 photographs of faces (20 disfigured, 20 nondisfigured), initially described by Ackerman et al. (2009). These were the same stimuli used by Miller and Maner (2011).

### Measuring attentional biases

We used a dot-probe task, experimental setup, and code identical to those used in the original study. On each trial, a face was displayed in one quadrant of the computer screen. After 500 ms, the face disappeared, and a categorization object (circle or square) was immediately presented in either the same location as the face (*congruent-location* trials) or a different quadrant (*incongruent-location* trials). Participants were instructed to respond as quickly as possible by categorizing the object as a circle (via the E key) or a square (via the I key). Each participant completed 80 trials in total (32 congruent-location trials and 48 incongruent-location trials). Each of the 40 faces (20 disfigured, 20 nondisfigured) was presented twice. Participants also completed 12 practice trials before completing those with faces. For these practice trials, items intended to be neutrally valenced and not associated with threats (infectious disease or otherwise; e.g., a mug, a pair of shoes, a spoon) were presented instead of faces.

Quadrant locations of faces and categorization objects were randomized, as were categorization-object shape and face types. These locations and shapes were constrained so that 32 trials used congruent locations and 48 trials used incongruent locations. As soon as the participant responded, the next trial started (i.e., the face in the next trial was presented immediately, and there was no intertrial interval). Trial order was randomized within each of the four blocks of trials.

Faces and categorization objects were presented at 20% of screen height, centered at 15% of screen height from the corner of each quadrant.

### Questionnaires

Self-report instruments were presented after the dot-probe task via Qualtrics surveys. Illness recency was assessed using both categorical and continuous measures. For the categorical measure, participants reported the last time they had a cold by choosing from the options “today,” “a couple days ago,” “a week ago,” “a couple weeks ago,” “a month ago,” “a few months ago,” or “a year or more ago.” Participants responding “today,” “a couple days ago,” or “a week ago” were categorized as recently ill, and all others were categorized as not recently ill.

For the continuous measure, participants responded to four statements on a 7-point scale (1 = *strongly disagree*, 7 = *strongly agree*): “Over the past couple of days, I have not been feeling well”; “Lately, I have been feeling a little under the weather”; “I have felt sick within the past week”; and “I had a cold or flu recently.” The average of these four scores was calculated. We note that in the original study, the interaction between face type and illness recency on reaction time was statistically significant (α = .05) when the categorical measure was used (*p* = .003) but not when the continuous measure was used (*p* = .08). However, the simple effect of relative attention toward disfigured faces was statistically significant for analyses using both the categorical and continuous measures (*p*s = .001 and .01, respectively, with relative attention estimated for participants 1 *SD* above the mean of the continuous illness-recency measure). Hence, following Miller and Maner (2011), we report outcomes of tests using both categorical and continuous measures. Given multiple tests of the same hypothesis, we used an alpha of .025 for both tests.

Participants also completed the Perceived Infectability and Germ Aversion subscales of the Perceived Vulnerability to Disease (PVD) scale (Duncan, Schaller, & Park, 2009). As outlined above, we administered other measures that were not reported by Miller and Maner (2011). These included the seven pathogen-disgust items from the Three Domain Disgust Scale (Tybur, Lieberman, & Griskevicius, 2009); ratings of images connoting infection risk developed by Curtis, Aunger, and Rabie (2004); and ratings of the faces used in the dot-probe task. For these tasks, participants rated items on a 7-point scale (1 = *not at all disgusting*, 7 = *extremely disgusting*). Finally, participants completed the HEXACO-60 (Ashton & Lee, 2009), a measure of the six HEXACO personality traits. Existing work suggests that perceptions of illness are partially influenced by personality (e.g., Feldman, Cohen, Doyle, Skoner, & Gwaltney, 1999). Using the HEXACO-60 allowed us to test whether the original results—if replicated—are independent of personality.

### Data-quality checks and data exclusions

For our primary analysis of the dot-probe task, we followed the original study by analyzing reaction times only from trials with correct responses 3 standard deviations of that participant’s mean reaction time. Participants with error rates greater than 3 standard deviations from the mean for the full sample were excluded from analyses. We also excluded these participants from the Three Domain Disgust Scale, PVD scale, and photo-rating analyses.

Other approaches to dot-probe data analysis, although not utilized in the study being replicated here, are defensible. We therefore also conducted exploratory analyses using other approaches (e.g., Winsorizing extreme responses and using reaction time to congruent trials as a covariate; see, e.g., McNulty, Meltzer, Makhanova, & Maner, 2018). Results from these analyses are reported in the Supplemental Material available online, as are findings from each individual data-collection site and findings modeling random effects for distractor stimuli.

### Analyses

Analysis R code is provided on OSF (https://osf.io/k2dbf/) and included in the Supplemental Material. We preregistered two types of analyses to evaluate the replication. The first tested the null hypothesis that the interaction between illness recency and face type is equal to zero. We preregistered our intention to test the simple effect of face type within the two categories of illness recency only if the null hypothesis were rejected. Mean reaction times were analyzed using an analysis of variance (ANOVA) with face type (disfigured, nondisfigured) as a within-subjects factor and illness recency as a between-subjects factor.

Results of replications that test only the null hypothesis of an effect size equal to zero can be ambiguous. Wide confidence intervals (CIs) can include both zero and the effect size found in the original study, and narrow confidence intervals can fail to overlap with zero but also be smaller than those included in the 95% CIs of the original study. To reduce such ambiguity, we also preregistered our intention to conduct an equivalence test, which treats the population parameter under the null hypothesis as a nonzero value. A rejected null indicates that the population effect size is unlikely to be equal to or greater than that value (Lakens, 2017). For replication studies, Simonsohn (2015) recommends testing an effect size that an original study had 33% power to detect. Samples 2.5 times larger than the original have roughly 80% power to reject population effect sizes of this magnitude.

The interaction described above is identical to an independent-samples *t* test on the differences in reaction times to the two face types between participants who had recently been ill and those who had not. Given that the original study had 28 participants who had recently been ill and 66 who had not recently been ill, we set the equivalence bound (*d*_z_) to 0.35—the effect size that the original design afforded 33% power to detect. Using the two one-sided tests procedure (Lakens, 2017), we interpreted *p* values below .05 as indicating that no meaningful effect exists, and we interpreted *p* values of .05 and above as indicating that the effect size (*d*_z_) could indeed be as high as 0.35.

We preregistered the same approach as Miller and Maner (2011) to test whether germ aversion, perceived infectability, disgust sensitivity, and disgust ratings of images vary across participants who were and were not recently ill. These analyses were not reported in Miller and Maner, but they tested conceptually similar hypotheses. All analyses were first conducted using the categorical illness-recency variable and then using continuous illness-recency variable. Hence, for each test, we used an alpha of .025 rather than .05.

## Results

On the basis of our preregistered exclusion criteria, we removed the 9 participants with error rates more than 3 standard deviations above the mean and two participants who did not complete both the questionnaire and the dot-probe task, resulting in a final sample of 402 participants, 151 of whom had recently been ill, and 251 of whom had not recently been ill. Alpha reliabilities were consistent with existing work for pathogen-disgust sensitivity (α = .69), germ aversion (α = .76), perceived infectability (α = .92), and ratings of disgust-eliciting images (α = .81). Average within-person response latencies following disfigured faces were highly correlated with average within-person response latencies following typical faces, *r* = .95 (Table 1).

**Table 1.** Descriptive Statistics for and Correlations Between Study Variables

Variable	*M*	*SD*	Correlations								
			1	2	3	4	5	6	7	8	9
1. Illness recency (discrete)	.38	.48	—	.73	.08	.13	.11	.12	.05	.11	.16
2. Illness recency (continuous)	3.44	1.67	[.69, .76]	—	.02	.05	.05	.09	.10	.08	.26
3. Latencies (difference; ms)	10.59	58.35	[−.04, .20]	[−.08, .12]	—	.43	.11	−.01	.01	−.02	−.02
4. Latencies (disfigured faces; ms)	644.49	179.58	[.00, .24]	[−.05, .15]	[.34, .50]	—	.95	.05	.03	.06	.08
5. Latencies (typical faces; ms)	633.90	163.50	[−.01, .22]	[−.05, .15]	[.01, .21]	[.94, .96]	—	.06	.03	.07	.09
6. Pathogen disgust	4.99	0.95	[.01, .23]	[−.00, .19]	[−.11, .09]	[−.04, .15]	[−.03, .16]	—	.60	.53	.17
7. Disgust toward Curtis images	4.34	1.16	[−.06, .16]	[.00, .19]	[−.09, .11]	[−.07, .13]	[−.07, .13]	[.53, .66]	—	.40	.14
8. Germ Aversion	3.86	1.11	[.01, .22]	[−.02, .17]	[−.12, .08]	[−.04, .16]	[−.03, .17]	[.45, .59]	[.32, .48]	—	.16
9. Perceived Infectability	3.65	1.28	[.05, .26]	[.17, .35]	[−.12, .07]	[−.02, .17]	[−.01, .19]	[.08, .27]	[.04, .23]	[.06, .25]	—

Note: Pearson correlations appear above the diagonal, and 95% confidence intervals appear below the diagonal. Point-biserial correlations derived from *t* values are reported between the dichotomous illness-recency variable and other variables.

**Fig. 1.**
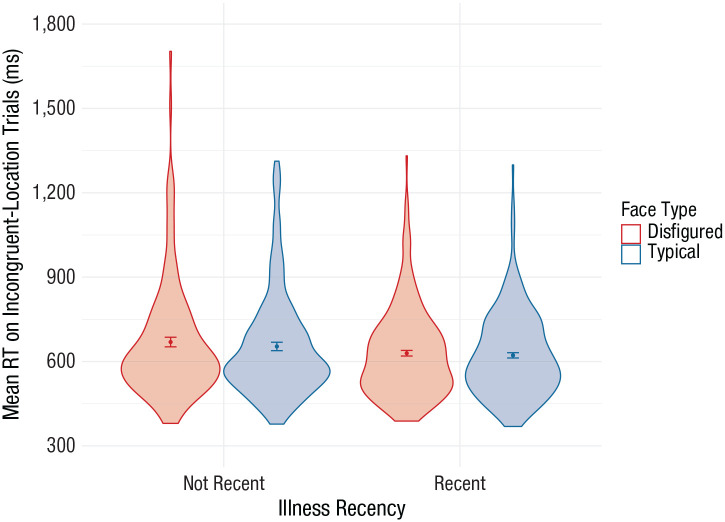
Mean reaction time (RT) on incongruent-location trials as a function of illness recency and face type. In each violin plot, the dot indicates the mean, errors bars indicate 95% confidence intervals, and the width of the shaded area indicates the distribution of the data.

We observed a main effect of face type: Responses were slower following disfigured faces (*M* = 644 ms, *SD* = 180) than typical faces (*M* = 634 ms, *SD* = 163), *F*(1, 400) = 14.96, η_*p*_^2^ = .036, 90% CI = [.009, .078], *p* < .001 (Fig. 1). The main effect of illness recency did not meet our preregistered threshold (*p* < .025)—recently ill: *M* = 661 ms, *SD* = 197; not recently ill: *M* = 626 ms, *SD* = 153, *F*(1, 400) = 4.23, η_*p*_^2^ = .010, 90% CI = [.000, .039], *p* = .040—nor did the interaction between illness recency and face type (disfigured vs. typical), *F*(1, 400) = 1.87, η_*p*_^2^ = .005, 90% CI = [.000, .027], *p* = .173. Whereas the original study detected attentional bias toward disfigured faces only for recently ill participants, we detected this same attentional bias for participants who were recently ill (*M* = 15.71 ms, 95% CI = [4.63, 26.79], *p* = .006) and for participants who were not recently ill (*M* = 7.51 ms, 95% CI = [1.19, 13.83], *p* = .02). We also did not detect an interaction between the continuous measure of illness recency and attentional biases toward disfigured relative to typical faces, *F*(1, 400) = 0.16, η_*p*_^2^ < .001, 90% CI = [0.000, 0.013], *p* = .691, and further, we did not detect a main effect of the continuous measure on response latencies, *F*(1, 400) = 1.06, η_*p*_^2^ = .003, 90% CI = [.000, .022], *p* = .303.

We were unable to reject the null hypothesis that illness recency is unrelated to attentional bias toward disfigured faces. But can we reject the null of the effect size (*d*_z_) being as large as ±0.35—what the original study had 33% power to detect? Yes—the 90% confidence intervals of the difference in attentional bias for participants who were and were not recently ill found here (*d*_z_ = −0.14, 90% CI = [−0.31, −0.04]) did not overlap with an effect size (*d*_z_) of −0.35, *t*(248.4) = 2.01, *p* = .023, or 0.35, *t*(248.4) = −4.55, *p* < .001.

We next examined relations between illness recency and the self-report measures. Relying on the categorical approach, and again using the preregistered critical *p* value of .025, we detected a relation between illness recency and perceived infectability, *r* = .16, 95% CI = [.05, .26], *p* = .006, but not between illness recency and germ aversion, *r* = .11, 95% CI = [.01, .22], *p* = .037; sensitivity to pathogen disgust, *r* = .12, 95% CI = [.01, .23], *p* = .038; or disgust ratings of the images from the Curtis data set, *r* = .05, 95% CI = [−.06, .16], *p* = .358. Results were similar using the continuous approach; we detected a relation between illness recency and perceived infectability, *r* = .26, 95% CI = [.17, .35], *p* < .001, but not germ aversion, *r* = .08, 95% CI = [−.02, .18], *p* = .127; sensitivity to pathogen disgust, *r* = .09, 95% CI = [−.00, .19], *p* = .062; or disgust ratings of images from the Curtis data set, *r* = .10, 95% CI = [.00, .18], *p* = .047. We also did not detect relations between illness recency and the six HEXACO factors (all *p*s > .07; see the Supplemental Material for full results, including alternative approaches to analyzing dot-probe data).

## Discussion

The study replicated here has been interpreted as a key piece of evidence supporting a relation between immunological resistance and pathogen avoidance in humans. Yet given the results from this direct replication, it should not be taken as evidence for such a relation. That said, because of two methodological limitations of the original study and this replication, we hesitate to interpret our null findings as strong evidence that pathogen avoidance does not vary as a function of immunological resistance. First, the dot-probe task has well-documented psychometric limitations, especially for the type of between-participant comparisons reported here (Parsons, Kruijt, & Fox, 2019). Second, the degree to which reporting having a head cold in the last 2 weeks reflects ability to resist pathogens is unclear. This latter shortcoming is perhaps shared by other studies that have seemingly demonstrated a relation between immunological resistance and pathogen avoidance, in which cocaine dependence (Ersche et al., 2014), progesterone (Fleischman & Fessler, 2011), rheumatoid arthritis (Oaten et al., 2017), trimester of pregnancy (Fessler, Eng, & Navarrete, 2005), and the same illness-recency measure used here (Miller & Maner, 2011, Study 2) have been interpreted as markers of a limited ability to resist pathogens. The use of such approaches has likely stemmed from the invasiveness and expense of measuring immune markers. But collaborations with controlled human-infection trials could allow for systematic control of the type of pathogen leading to infection and the time course of infection, as well as pre- and postinfection observations.

We detected a relation that the original study did not: Illness recency related to the PVD scale’s Perceived Infectability subscale. Rather than reflecting a shift in pathogen avoidance when immunological resistance is low, though, this relation indicates that reports of recent illness covary with reports of general illness frequency. Illness recency was unrelated to the other PVD subscale (Germ Aversion), disgust sensitivity, and disgust ratings of images of pathogen threats. Variables that have been deployed interchangeably to test behavioral-immune-system hypotheses (e.g., attentional bias toward disfigured faces in a dot-probe task, disgust sensitivity, germ aversion, and perceived infectability; see Tybur, Frankenhuis, & Pollet, 2014) had correlations (*r*s) between −.02 (attentional bias and germ aversion) and .60 (disgust sensitivity and disgust ratings of images). Notably, we did not detect a relation between attentional bias toward disfigured faces and disgust sensitivity or germ aversion; this might suggest that the main effect arose from low-level features of the disfigured faces (e.g., coloration) rather than from the stimuli being interpreted as infectious. This research area would benefit from better developing the validity of the many measures deployed here. Such an endeavor would match calls to view the behavioral immune system as just that—a modular system with distinct components, not all of which can or should respond identically to something such as capacity to resist infection (Gangestad & Grebe, 2014).

## Concluding Thoughts

The existence of modular features of human psychology dedicated to neutralizing pathogens is not in question (e.g., Ackerman et al., 2018; Curtis et al., 2004; Schaller & Park, 2011; Tybur & Lieberman, 2016). A key task for psychologists is understanding the nature of this psychology and, hopefully, using it to better understand topics ranging from food choice to intergroup relations to health decisions, among other things. We hope that this preregistered direct replication will not be perceived as repudiating a key hypothesis in this area but rather will facilitate progress in the substantial and growing literature on the psychological underpinnings of pathogen avoidance.

## Supplemental Material

Tybur_Supplemental_Material_rev – Supplemental material for Preregistered Direct Replication of “Sick Body, Vigilant Mind: The Biological Immune System Activates the Behavioral Immune System” Click here for additional data file. Supplemental material, Tybur_Supplemental_Material_rev for Preregistered Direct Replication of “Sick Body, Vigilant Mind: The Biological Immune System Activates the Behavioral Immune System” by Joshua M. Tybur, Benedict C. Jones, Lisa M. DeBruine, Joshua M. Ackerman and Vanessa Fasolt in Psychological Science

## Transparency

*Action Editor:* D. Stephen Lindsay

*Editor:* D. Stephen Lindsay

*Author Contributions*

All five authors contributed to writing the manuscript. B. C. Jones and L. M. DeBruine generated the analysis script, and L. M. DeBruine and V. Fasolt analyzed the data. All authors approved the final version for submission.

**Open Practices:** All data and analysis code have been made publicly available via OSF and can be accessed at https://osf.io/k2dbf/. The design and analysis plans for this study were preregistered at https://osf.io/k2dbf/. This article has received the badges for Open Data and Preregistration. More information about the Open Practices badges can be found at http://www.psychologicalscience.org/publications/badges.
