# Supplementary material for: Preregistered Direct Replication of “Sick Body, Vigilant Mind: The Biological Immune System Activates the Behavioral Immune System”
Source: Psychol Sci. 2020 Oct 20;31(11):1461–9. doi: 10.1177/0956797620955209 (PMC7675771; doi:10.1177/0956797620955209)
Supplement: Tybur_Supplemental_Material_rev – Supplemental material for Preregistered Direct Replication of “Sick Body, Vigilant Mind: The Biological Immune System Activates the Behavioral Immune System” [file Tybur_Supplemental_Material_rev.docx]

Preregistered replication of “Sick body, vigilant mind:

The biological immune system activates the behavioral immune system”

SUPPLEMENTAL MATERIALS

Joshua M. Tybur^1, 2^

Benedict C. Jones^3^

Lisa M. DeBruine^3^

Joshua M. Ackerman^4^

Vanessa Fasolt^3^

1. VU Amsterdam

2. Institute Brain and Behavior Amsterdam

3. Institute of Neuroscience & Psychology, University of Glasgow, UK

4. University of Michigan

ALTERNATIVE APPROACHED TO DOT PROBE ANALYSES

We used three alternative approaches to analyzing the dot probe data. First, we conducted further analyses by windsorizing extreme responses (i.e., transforming within-participant responses greater than 3SD from the participant-specific mean to be 3SD from that participant’s mean) rather than removing them. Results were unchanged; again, attentional bias toward disfigured faces did not vary as a function of categorical illness recency, *F*(1, 400) = 2.63, *p* = .11, or continuous illness recency *F*(1,400) = 0.63, *p* = .43. Second, we conducted an analysis in which reaction times following healthy faces was treated as a covariate rather as a level of a repeated measures factor. Here, too, results were unchanged; bias toward disfigured faces did not vary as a function of illness recency, F(1, 399) = 1.36, p = 0.244. Third, we analyzed trial-level reaction times used mixed effects modeling, with random intercepts modeled for participants, testing sites, and stimulus (i.e., face identity). Here too there was no interaction between face type and categorical illness recency, *t*(17886) = -1.35, *p* = .178 or between face type and continuous illness recency, *t*(17886) = -0.45, *p* = .647.

DOT PROBE RESULTS AT INDIVIDUAL SITES

We also analyzed the dot probe data separately at each site. Using the categorical measure of illness recency, attentional bias did not vary across recently ill and not-recently-ill participants at Ann Arbor, *F*(1, 138) = 0.53, *p* = .467, Amsterdam, *F*(1, 142) = 0.01, *p* = .908, or Glasgow, *F*(1, 116) = 1.26, *p* = .263. Similarly, the continuous measure of illness recency was unrelated to attentional bias in Ann Arbor, *F*(1, 138) = .08, *p* = .775, Amsterdam, *F*(1, 142) = .12, *p* = .727, or Glasgow, *F*(1, 116) = .03, *p* = .875.

ILLNESS RECENCY AND HEXACO PERSONALITY

| hexaco | not recent | recent | t.value | p.value | df |
| --- | --- | --- | --- | --- | --- |
| H | 3.44 | 3.33 | 1.7800 | 0.0764 | 312 |
| E | 3.47 | 3.48 | -0.1150 | 0.9080 | 314 |
| X | 3.21 | 3.20 | 0.0438 | 0.9650 | 303 |
| A | 3.21 | 3.22 | -0.0810 | 0.9360 | 301 |
| C | 3.67 | 3.70 | -0.4140 | 0.6790 | 310 |
| O | 3.57 | 3.54 | 0.3890 | 0.6980 | 334 |
